# The Expanding Role of Cancer Stem Cell Marker ALDH1A3 in Cancer and Beyond

**DOI:** 10.3390/cancers15020492

**Published:** 2023-01-13

**Authors:** Meghan E. McLean, Maya R. MacLean, Hannah F. Cahill, Raj Pranap Arun, Olivia L. Walker, Marie-Claire D. Wasson, Wasundara Fernando, Jaganathan Venkatesh, Paola Marcato

**Affiliations:** 1 Department of Pathology, Dalhousie University, Halifax, NS B3H 4R2, Canada; 2 Department of Microbiology and Immunology, Dalhousie University, Halifax, NS B3H 4R2, Canada; 3 Nova Scotia Health Authority, Halifax, NS B3H 4R2, Canada

**Keywords:** ALDH1A3, cancer stem cells, chemoresistance, glycometabolism, cell signaling, gene expression, diabetes, inhibitors

## Abstract

**Simple Summary:**

Aldehyde dehydrogenase 1A3 (ALDH1A3) is the primary cause of the high ALDH activity associated with cancer stem cell (CSC) populations in multiple cancers and its expression promotes cancer progression. Here in, we review the role of ALDH1A3 in normal physiology, cancer, and other diseases. Finally, we discuss the emerging potential of targeting ALDH1A3 with non-specific and specific inhibitors.

**Abstract:**

Aldehyde dehydrogenase 1A3 (ALDH1A3) is one of 19 ALDH enzymes expressed in humans, and it is critical in the production of hormone receptor ligand retinoic acid (RA). We review the role of ALDH1A3 in normal physiology, its identification as a cancer stem cell marker, and its modes of action in cancer and other diseases. ALDH1A3 is often over-expressed in cancer and promotes tumor growth, metastasis, and chemoresistance by altering gene expression, cell signaling pathways, and glycometabolism. The increased levels of ALDH1A3 in cancer occur due to genetic amplification, epigenetic modifications, post-transcriptional regulation, and post-translational modification. Finally, we review the potential of targeting ALDH1A3, with both general ALDH inhibitors and small molecules specifically designed to inhibit ALDH1A3 activity.

## 1. Introduction

In 2011, the then little-studied aldehyde dehydrogenase 1A3 (ALDH1A3) enzyme in the context of cancer, was shown to generate the high Aldefluor activity associated with breast cancer stem cells (CSCs) [1]. Since then, interest in investigating the roles of ALDH1A3 in cancer, and the potential of targeting ALDH1A3, have grown exponentially. Additionally, ALDH1A3 plays critical roles in other diseases, such as in the development of type 2 diabetes, where dysregulation of ALDH1A3 in pancreas β-cells impairs insulin production. This review provides a broad overview of ALDH1A3 function in both normal and disease contexts. We discuss its expanding modes of action and the mechanisms of ALDH1A3 regulation in cancer. Finally, the development of increasingly more specific ALDH1A3 inhibitors suggests the potential of clinically targeting ALDH1A3 is becoming more likely.

## 2. ALDH1A3 Is a Member of the ALDH Superfamily

Found to be generally expressed at low levels in the body, with higher amounts in the salivary gland, stomach, and kidneys, ALDH1A3 was the sixth ALDH enzyme discovered in the human genome and was initially called ALDH6 [2]. Eventually, 19 ALDH enzymes expressed from the distinct genetic loci in the human genome would be discovered. The 19 members comprise the ALDH superfamily and share at least 40% sequence homology, with subfamily members sharing at least 60% homology [3,4].

ALDHs catalyze the irreversible oxidation of aldehydes to carboxylic acids by binding an aldehyde and cofactor nicotinamide adenine dinucleotide (NAD^+^) or NAD phosphate (NADP^+^). In general, ALDHs function to remove toxic aldehydes generated during metabolic processes, including endogenous aldehydes that arise from lipid peroxidation, amino acid catabolism, and exogenous xenobiotics [4]. In addition, the isoforms have distinct expression profiles in body tissues, differing subcellular locations (cytoplasm, nucleus, endoplasmic reticulum, or mitochondria), substrate specificity, and function. Pertinent to this review, the homologous ALDH1A1, ALDH1A2, and ALDH1A3 isoforms share 70% amino acid sequence homology, are cytoplasmic, and oxidize the vitamin A metabolite all-trans retinal to all-trans retinoic acid (ATRA, also commonly referred to as retinoic acid, RA). Due to this retinal oxidizing activity, ALDH1A1, ALDH1A2, and ALDH1A3 are also called retinal dehydrogenase 1 (RALDH1), RALDH2, and RALDH3, respectively. 

The three ALDH1A enzymes have important and distinct roles in embryonic development. ALDH1A3 is expressed in the ventral retina and its loss causes anophthalmia and aberrant eye development in humans and animal models [5,6,7]. ALDH1A3 knockout in mice is neonatal-lethal, with severe defects in nasal and eye development, due to RA deficiency during critical developmental periods [8].

A comparative analysis of the three ALDH1A enzymes revealed similar structural topologies, with ALDH1A3 having the smallest substrate-binding pocket [8]. ALDH1A3 had the highest enzymatic activity for the conversion of all-trans-retinal to RA, followed by ALDH1A2, but comparatively had the least activity with other tested substrates [8]. This was consistent with earlier reports suggesting the greater RA biosynthetic capacity of ALDH1A3 over ALDH1A1 [9].

## 3. Retinoic Acid Signaling Is a Key Function of ALDH1A Enzymes

RA is a developmentally important cell signaling molecule; it is a ligand for the nuclear hormone receptor retinoic acid receptor (RAR), capable of regulating the expression of hundreds of genes and resulting in diverse cellular effects [4,10,11,12]. A requisite of RA signaling is that cells can metabolize vitamin A (retinol) to retinal and then retinal to RA. 

RA binds to the nuclear hormone receptors retinoic acid (RAR) α, β, γ, which form heterodimers with the retinoid X receptor (RXR) [13]. The heterodimers regulate gene expression by binding to retinoic acid receptor element (RARE) sequence motifs found in the promoters and enhancer regions of over 3000 genes in the genome [14]. The binding of RA to these heterodimer nuclear hormone receptors can have both activating and repressing gene expression effects. Gene induction is observed when RA binding to RAR promotes the binding of coactivators of the nuclear receptors and other coactivators such as histone acetylases (HATs). Inversely, in a mechanism that is less understood, gene repression by RA involves RA-mediated recruitment of polycomb repressive complex 2 (PRC2) and superfamily histone deacetylase (HDAC) to nuclear hormone receptor heterodimers. 

RA signaling in the physiological range (nM amounts) is mediated by the ALDH1A enzymes and has distinct effects from the supra-physiological effects induced by pharmacological RA treatment, which is in the µM range. Supra-physiological amounts of RA can inhibit cell proliferation and induce cell death and differentiation, as seen in the treatment of acute promyelocytic leukemia [15,16,17]. RA treatment can reduce the severity of asthma [18,19], while in contrast, retinoid and vitamin A deficiency exacerbate the condition [20,21]. Gene expression analysis indicated elevated ALDH1A3 expression in asthma patients [22]; however, another study showed no change in ALDH1A3 protein expression levels [19]. 

Overall, these studies suggest that in the context of cancer and other illnesses, pharmacological retinoid treatment effects often differ from ALDH1A-mediated physiological RA signaling and the two should not be necessarily equated.

## 4. ALDH1A3 Is a Cancer Stem Cell Marker

Cancer stem cells (CSCs) are a small subpopulation of cells within tumors that exhibit characteristics of both stem cells and cancer cells. CSCs are enriched for various markers, with some cancer-type specificity. These markers include cell surface markers such as CD133, CD24, CD44, and epithelial cell adhesion molecule (EpCAM) [23]. Among the most common methods to identify cancer cell populations enriched for CSCs is increased ALDH activity detected by the Aldefluor assay [24]. Aldefluor-positive (also referred to as ALDH^bright^ or ALDH^high^) populations were initially identified as having CSC qualities (i.e., having increased tumorigenicity and giving rise to heterogeneous tumors) in murine xenograft studies with breast cancer by Ginestier et al., [25] and leukemia by Cheung et al., [26]. Aldefluor-positive-isolated cancer cells have been similarly shown to generate xenograft tumors with high efficiency in the liver, head and neck, lung, pancreatic, cervical, thyroid, prostate, colon, bladder, and ovarian cancers [27,28,29,30,31,32,33,34,35]. 

The Aldefluor assay measures the conversion of ALDH substrate, BODIPY™ amino acetaldehyde, to fluorescent reaction product BODIPY™ aminoacetate. The addition of inhibitor diethylaminobenzaldehyde (DEAB) reduces fluorescence, confirming that Aldefluor-positive cells are correctly identified. This assay was originally developed for the isolation of viable hematopoietic stem cells from human umbilical cord blood [36] and was initially believed to be specific for one ALDH isoform found in high abundance in those cells: ALDH1A1. Therefore, Aldefluor-positive cells are sometimes referred to as ALDH1 positive or ALDH1A1 positive. This can be a wrong assumption since the BIODIPY aminoactealdehyde substrate is not specific to ALDH1A1 and other ALDH enzymes can generate the fluorescent product if expressed in sufficient levels [24].

Multiple studies have demonstrated that ALDH1A3 is an ALDH isoform that is at least as important as ALDH1A1 in influencing the Aldefluor activity of cancer cells. For breast cancer, gene expression analysis and knockdown of the 19 ALDH isoforms revealed that ALDH1A3 expression was the primary isoform contributing to Aldefluor activity of breast cancer patient tumors and cell lines [1]. Later, similar studies performed in melanoma cancer implicated both ALDH1A1 and ALDH1A3 expression as being important in determining Aldefluor activity and CSC activity [37]. Similarly, in mesenchymal glioma stem cells, Aldefluor-positivity was associated with enriched ALDH1A3 expression and stemness [38]. Profiling the ALDH isoforms by gene expression and knockdown in non-small cell lung cancer similarly revealed the importance of ALDH1A3 in the Aldefluor activity of cancer and tumorigenicity [39]. In colon cancer, analysis of expression and knockdown of the 19 ALDH isoforms in 58 cell lines again suggested the primary importance of ALDH1A3 in the Aldefluor activity colon [40]. In intrahepatic cholangiocarcinoma (bile duct cancer) ALDH1A3 was found to be the main contributor to Aldefluor activity [41]. In head and neck cancer, ALDH activity and stemness were associated with ALDH1A3 expression [42]. ALDH1A3 imparts stemness, tumorigenicity, and Aldefluor activity in gastric cancer [43]. In addition to highlighting the role of ALDH1A3 in the Aldefluor activity of multiple cancers, these studies also demonstrate that when identifying CSCs, detecting the expression of ALDH enzymes is not equal to performing the Aldefluor assay [44]. 

It is important to note that ALDH1A3 is also commonly measured by many other methods, including immunohistochemistry and immunofluorescence, Western blotting, RNA sequencing, and quantitative polymerase chain reaction (QPCR). Many of the subsequent studies we discuss detect and quantify ALDH1A3 in cells and tissues by these other methods.

## 5. ALDH1A3 Is Associated with Worse Prognosis in Cancer

Consistent with ALDH1A3 association with CSCs, ALDH1A3 expression in cancer is generally associated with worse outcomes, progressive disease, and recurrence. In breast cancer, patient tumors with high levels of ALDH1A3 were associated with an increased incidence of metastasis compared to those with low levels of ALDH1A3 [1]. ALDH1A3 is higher in triple-negative breast cancer (TNBCs), which is an aggressive subtype of breast cancer [45]. In TNBC, ALDH1A3 is associated with worse survival. In addition, high ALDH1A3 expression is associated with worse patient survival in prostate, glioblastoma, neuroblastoma, pancreatic, gastric, gall bladder, colon, and intrahepatic cholangiocarcinoma cancers [41,45,46,47,48,49,50,51,52]. High levels of ALDH1A3 are correlated with increased tumor grade in breast, glioblastoma, bladder, and prostate cancer [45,46,47,53]. Bladder cancer, breast cancer, and intrahepatic cholangiocarcinoma were shown to have increased ALDH1A3 expression along with high tumor stage [41,45,53]. 

Although ALDH1A3 is associated with increased tumor progression and worse prognosis in many cancer types, increased ALDH1A3 expression has also been associated with better patient outcomes in TP53 wildtype ovarian tumors, BRAF-mutated metastatic melanoma, and non-small cell lung cancer [39,54,55]. These positive clinical correlates with ALDH1A3 in a different context suggest that ALDH1A3 effects in cancer could be cellular context-specific and dependent on the presence of other molecular factors.

## 6. ALDH1A3 Promotes Tumor Progression

The association of ALDH1A3 with CSCs in multiple cancers implies its importance to cancer progression and aggressiveness. Indeed, ALDH1A3 can facilitate cancer progression by promoting tumor growth and metastasis, and these effects are mirrored in vitro assays across multiple cancers.

Knockdown of ALDH1A3 inhibited the growth of the glioma Aldefluor-positive cells, suggesting that ALDH1A3 contributes to CSC-mediated tumorigenicity of mesenchymal glioma [38]. In melanoma cells, ALDH1A3 knockdown reduced tumor growth activity [37]. In non-small cell lung cancer, tumorigenicity was reduced upon ALDH1A3 knockdown [39]. In breast cancer, the effects of ALDH1A3 were not as clear, with ALDH1A3 promoting tumor growth in two triple-negative breast cancer cell lines (MDA-MB-231 and MDA-MB-435 cells) but inhibiting in a third (MDA-MB-468 cells) [45]. The mechanism behind this discrepancy may be related to cell line-specific differential epigenetic-silencing of key ALDH1A3-inducible genes, including mucin 4 (MUC4) and homeobox A1 (HOXA1). In gastric cancer, ALDH1A3 knockdown reduced tumor growth [51]. In osteosarcoma, tumorigenicity was associated with ALDH1A3 expression [56].

ALDH1A3 also contributes to metastasis. In TNBC MDA-MB-231 cells, increased ALDH1A3 resulted in a corresponding increase in lung metastasis in the orthotopic xenograft model [45]. Knockdown of ALDH1A3 in HPAC pancreatic cancer cells reduced resulting lung metastasis when tail-vein-injected into mice [52]. ALDH1A3 has been linked to pancreatic cancer metastasis. 

In vitro analyses suggests that ALDH1A3’s effects on tumor growth and metastasis are multifactorial. ALDH1A3 knockdown melanoma cell lines resulted in decreased cell proliferation and increased apoptosis [37,57]. In colon cancer cell lines, ALDH1A3 knockdown decreased cell proliferation and C-X-C chemokine receptor type 4 (CXCR4) expression, suggesting a potential connection between the two [40]. In lung cancer cell lines, reduced ALDH1A3 expression was associated with decreased cell proliferation [58]. In gastric cancer cells, ALDH1A3 knockdown reduced cell proliferation [51]. 

Although the increased metastasis associated with increased ALDH1A3 could be an indirect result of increased tumor burden and cancer cell proliferation, there is also evidence that ALDH1A3 directly increases the metastatic potential of a cancer cell. There are many reports of ALDH1A3 affecting invasion and/or migration, but these effects appear cancer-type dependent. For breast cancer, increased ALDH1A3 results in increased transwell invasion of TNBC MDA-MB-231 cells [45]. The increased invasion/metastatic potential imparted by ALDH1A3 on breast cancer cells appears connected to decreased migration. ALDH1A3 knockdown in TNBC MDA-MB-468 and SUM159 cells increased adhesion and migration while decreasing metastasis in a chick chorioallantoic membrane assay [59]. ALDH1A3 knockdown in cholangiocarcinoma bile duct cancer cell lines decreases migration [41]. 

Reports also suggest that ALDH1A3 imparts increased colony formation or clonogenicity, which measures the ability of a single cell to form a colony, an in vitro indicator of the tumor-initiating capacity required to form primary and secondary tumors [60]. In a panel of lung cancer cell lines, ALDH1A3 knockdown reduced colony formation in 11 out of 12 cell lines [39]. In breast cancer, ALDH1A3 imparted increased colony formation to TNBC MDA-MB-231 and MDA-MB-468 cells [61]. Similarly, in colon and gastric cancers, reduced ALDH1A3 resulted in decreased colony formation [51,62]. In neuroblastoma, ALDH1A3 knockdown reduced clonogenicity [48]. 

In summary, ALDH1A3 promotes tumor progression, likely via effects on proliferation, apoptosis, migration, invasion, and clonogenicity. The accumulating evidence of ALDH1A3 as a key factor in cancer progression across multiple cancer types suggests it is a promising therapeutic target. Current advances in targeting ALDH1A3 will be discussed later in this review.

## 7. The Role of ALDH1A3 Multiple Drug Resistance

The role of CSCs, and by extension CSC markers and ALDH activity, in mediating drug tolerance has been extensively reported and has expanded our understanding of the mechanisms leading to chemoresistance. CSCs exhibit multiple drug resistance (MDR) through many mechanisms, including increased expression of ATP-binding cassette (ABC) transporters and dysregulation of signaling pathways that govern drug resistance (e.g., Hippo/Yap/Taz, Wnt, Notch, JAK/STAT and Hedgehog pathways) [63,64]. 

MDR associated with CSCs has also been ascribed through effects mediated by various CSC markers. For example, the CSC EpCAM promotes MDR in breast cancer by inducing partial epithelial to mesenchymal transition (EMT) [65]. In leukemia EpCAM+ cells, chemoresistance was a consequence of increased WNT5a signaling. The contributions of the various isoforms in mediating this phenotype have been assessed and reviewed elsewhere [63,66]. Here in, we focus on the specific role of ALDH1A3 in impacting MDR in cancer.

ALDH1A3 is highly expressed in therapy-resistant cancer cell subpopulations and has been shown to confer resistance to several chemotherapeutic agents, including doxorubicin, paclitaxel, docetaxel, temozolomide, 5-fluorouracil, oxaliplatin, and cisplatin in multiple cancers, such as colorectal cancer [64,67], lung adenocarcinoma [68], melanoma [37], malignant pleural mesothelioma [69,70], gastric cancer [51], prostate cancer [71], osteosarcoma [56], colon cancer [72], and human embryonal carcinoma cells [73]. In these studies, knocking down or inhibiting ALDH1A3 sensitized the cancer cells to the chemotherapies, illustrating that ALDH1A3 mediates chemoresistance in cancer.

In studies where the mechanism of ALDH1A3-mediated chemoresistance was analyzed, it seems to not be a direct mechanism where ALDH1A3 inactivates the drugs, but an indirect mechanism, where ALDH1A3-induced signaling and gene expression changes result in chemoresistance. It is noteworthy that this is distinct from other ALDH enzymes, such as ALDH1A1 and ALDH3A1, where there is evidence of enzymatic drug detoxification, such as in the metabolism of cyclophosphamide leading to cyclophosphamide resistance [74,75,76]. 

**Figure 1:**
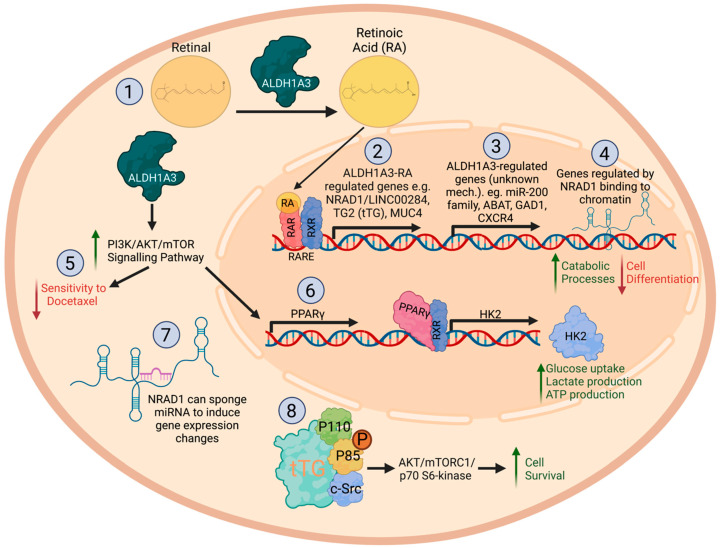
Cancer-promoting mechanisms of ALDH1A3. (1) ALDH1A3 can oxidize retinal to retinoic acid (RA) and [4] (2) RA induces gene expression changes by binding heterodimers of RAR and RXR, which are bound to RARE sequences in gene promoter regions [13]. RA-inducible genes include NRAD1 (LINC00284), TG2, MUC4. (3) ALDH1A3 also regulates the expression of many other genes through unknown mechanisms leading to cancer-promoting effects [40]. (4) ALDH1A3 regulates gene expression indirectly through NRAD1, an inducible gene by the ALDH1A3-RA pathway. NRAD1 binds chromatin leading to the regulation of genes enriched in biological processes that regulate catabolism and differentiation [77]. (5) Through ALDH1A3 the PI3K/AKT/mTOR signaling pathway becomes upregulated, which leads to a decrease in docetaxel sensitivity and [71] (6) PPARγ levels [52]. PPARγ forms a heterodimer with RXR and increases HK expression. HK increases glucose uptake, lactate production, and ATP production. (7) ALDH1A3-regulated NRAD1 can sponge miRNAs and therefore lead to gene expression changes [77]. (8) Through the ALDH1A3-RA pathway, tTG (encoded by TG2) binds to p110, p85, and c-Src to induce the AKT/mTORC1/p70 S6-kinase pathway and increases cell survival [78]. This figure was created with BioRender.com.

In contrast to direct effects on drug metabolism, we observe cell signaling mediated effects by ALDH1A3 in cancer cells leading to MDR. In prostate cancer, increased ALDH1A3 activated the phosphatidylinositol 3-kinase/Protein kinase B/rapamycin (PI3K/AKT/mTOR) signaling pathway conferred a survival advantage to prostate cancer cells and decreasing their sensitivity to docetaxel (Figure 1) [71]. Similarly, in gastric cancer, ALDH1A3-mediated 5-fluorouracil resistance was connected to ALDH1A3 effects on gene expression of the mammalian target of mTOR and phosphorylation of mTOR target S6 kinase [51].

Other reports connect ALDH1A3 dysregulation to chemoresistance. In cholangiocarcinoma cells, upregulation of the epidermal growth factor receptor (EGFR) and the consequent activation of the Signal Transducer and Activator of Transcription 3 (STAT3) and extracellular signal-regulated kinases (Erk) signaling pathways increased ALDH1A3 expression and resulted in gemcitabine resistance [79]. Similarly, in malignant pleural mesothelioma, the STAT3- nuclear factor-kappa B/DNA Damage Inducible Transcript 3/enhancer-binding protein beta (STAT3-NF-kB/DDIT3/CEBPβ) axis was demonstrated to regulate ALDH1A3 expression and reduce sensitivity to pemetrexed + cisplatin treatment [69]. Inhibition of STAT3-NFkB activity decreased ALDH1A3 expression by preventing the binding of transcription factor CEBPβ to its promoter, consequently restoring chemotherapy sensitivity. In gastric cancer, ALDH1A3 causes 5-fluorouracil and cisplatin resistance in connection with the histone demethylase and oncogene Lysine Demethylase 4C (KDM4C). KDM4C activates ALDH1A3 expression by reducing the epigenetic modifications H3K9me2 and H3K9me3 at its promoter. In turn, ALDH1A3 increases KDM4C levels, thereby establishing a KDM4C-ALDH1A3 feedforward regulation which results in chemoresistance in gastric cancer [43]. 

ALDH1A3-associated chemotherapy resistance across cancer types is consistent with its general importance in tumor progression and associations with CSCs and worse prognosis. Additionally, ALDH1A3 expression was reported to be enriched in EGFR-mutated non-small cell lung carcinoma cells resistant to the EGFR tyrosine kinase inhibitor erlotinib [80], suggesting that ALDH1A3-expressing cancer cells are resistant to other treatments beyond chemotherapy. These treatments include radioresistance, as was shown for head and neck cancer [42]. The fact that ALDH1A3 knockdown sensitizes cancer cells to therapy [37,41,51,64,68,69] further supports the need for the development of clinical ALDH1A3 inhibitors as adjuvant therapies (discussed later in this review).

## 8. Regulation of ALDH1A3 in Cancer

**Figure 2:**
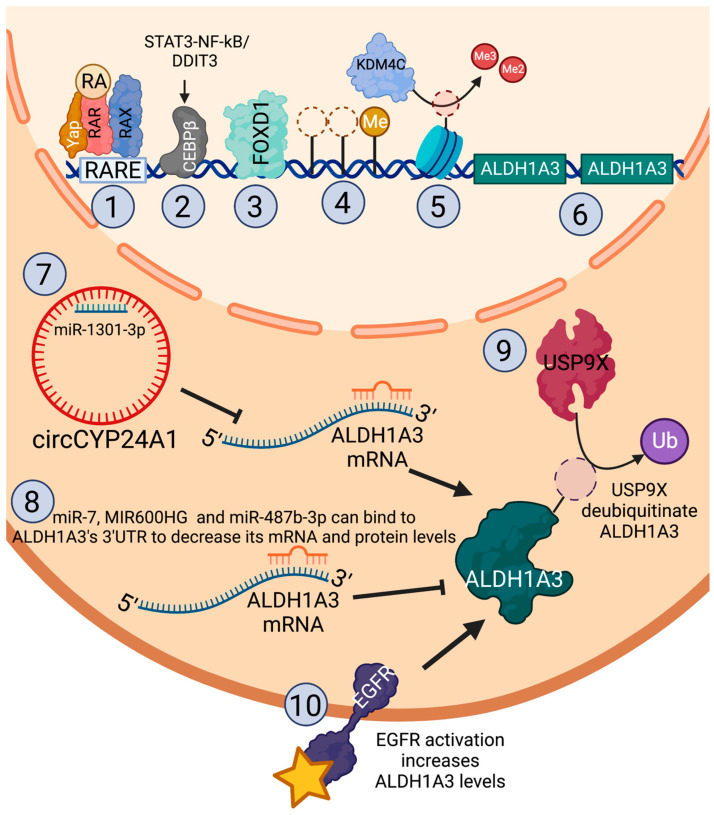
Mechanisms of regulation of ALDH1A3 in cancer. ALDH1A3 is transcriptionally regulated by (1) Yap-activated RAR-RXR [67], and (2) transcription factors CEBPβ [69], (3) FOXD1 [81]. ALDH1A3 is epigenetically regulated by (4) promoter DNA methylation [83] and (5) histone methylation by KDM4C [43]. (6) The ALDH1A3 gene is also amplified in some cancers, as summarized in Table 1. ALDH1A3 is post-transcriptionally regulated by non-coding RNAs via (7) circCYP24A1 [71], which sponges miR-1301-3p, preventing its binding and targeting of the ALDH1A3 mRNA, or by (8) miR-7, MIR600HG, and miR-487b-3p, which target the ALDH1A3 transcript through binding sequences in its 3′UTR [56,72,88]. ALDH is regulated post-translationally by (9) USP9X, which deubiquitinates ALDH1A3, inhibiting its degradation [82]. ALDH1A3 is regulated indirectly by other signaling pathways, such as (10) activation of EGFR, which leads to increased ALDH1A3 levels [79]. This figure was created with BioRender.com.

ALDH1A3 upregulation in cancer occurs via multiple mechanisms (Figure 2). As described in the above section, The role of ALDH1A3 in multiple drug resistance section, transcriptional ALDH1A3 upregulation in cancer has been connected to histone epigenetic modification of its promoter [43], indirect upregulation through EGFR [80], or STAT3-NF-kB [69], and the activity of transcription factor CEBPβ [69]. Similarly, in mesenchymal glioma stem cells, transcription factor foxhead box D1 (FOXD1) regulates ALDH1A3 its expression, and the FOXD1-ALDH1A3 axis is critical in the self-renewal and tumorigenicity properties of glioma stem cells [81]. ALDH1A3 levels are also dysregulated at the protein level in glioma stem cells, where ubiquitin-specific protease 9X (USP9X) deubiquitinates ALDH1A3, leading to its increased stabilization and levels [82]. 

In addition to the above-described epigenetic regulation by histone modification, DNA methylation of the ALDH1A3 promoter leads to its reduced expression of ALDH1A3 in glioblastoma [83,84]. In both these studies, ALDH1A3 hypermethylation was associated with better prognostic outcomes, consistent with ALDH1A3′s caner-promoting role. In contrast, in bladder cancer, ALDH1A3 hypermethylation was associated with progressive disease and recurrence in non-muscle invasive bladder cancer [53].

As described in the Retinoic acid signaling—a key function of ALDH1A enzymes section, ALDH1A3 produces RA, which is a ligand for nuclear hormone RAR-RXR heterodimers, leading to transcriptional regulation of genes with RAREs. ALDH1A3 is also transcriptionally regulated by RA and RAR-RXR through feedback loop regulation [67]. A pan-RAR antagonist inhibited ALDH1A3 expression [67]. In colorectal cancer cells, ALDH1A3 expression is induced via the Hippo pathway activator Yap by its RAR-RXR transcriptional coactivator activity [67]. 

ALDH1A3 is also commonly regulated via post-transcriptional regulation by non-coding RNAs, both long non-coding RNA (lncRNA) and microRNA (miRNA). LncRNAs are over 200 nucleotides in length and regulate gene expression through multiple mechanisms, including chromatin modification, scaffolding, protein, and RNA binding [85]. In contrast, the 18–25 nucleotide miRNAs negatively regulate target mRNA stability and translation [86]. Crosstalk and interactions between lncRNAs and miRNAs further influence gene expression [87].

In prostate cancer, circular RNA circCYP241 indirectly upregulates ALDH1A3 by sponging microRNA miR-1301-3p, which targets ALDH1A3 transcripts [71]. Similarly, in breast cancer cells, ALDH1A3 expression is suppressed cells through miR-7 by binding a sequence in the 3′ untranslated region (UTR) [88]. Introduced miR-7 expression in breast cancer cells reduced tumor growth and CSC features. In osteosarcoma, miR-487b-3p targets ALDH1A3, leading to chemosensitivity and reduced CSCs [56]. In colorectal cancer, ALDH1A3 expression is targeted by long non-coding RNA (lncRNA) MIR600HG by binding to a sequence in its 3′UTR, resulting in reduced metastasis and chemosensitivity [72].

Finally, we surveyed the available patient tumor datasets for cBioportal in cancers that ALDH1A3 has been implicated in tumor progressions for potential mutation mechanisms leading to changes in gene expression. Gene amplification was consistently the most reported ALDH1A3 mutation, and this would lead to increased levels of ALDH1A3 in those cancers (Table 1). Notably, amplification of ALDH1A3 was more common in TNBCs among all breast cancer subtypes, which is consistent with its increased expression in TNBCs. Further, consistent with ALDH1A3′s role in metastasis, increased incidence of ALDH1A3 gene amplification occurred in datasets of metastatic prostate, breast, and melanoma (Table 1). In contrast, a few instances of deletion would lead to reduced expression or mutations that would cause truncation or impair protein function.

Together, the published studies and dataset analysis suggest that ALDH1A3 expression dysregulation in cancer is multifactorial and mediation by genetic, epigenetic, post-transcriptional, and post-translational mechanisms. 

## 9. Mechanisms of ALDH1A3 in Cancer: Effects on Gene Expression

As detailed in the section Retinoic acid signaling—a key function of ALDH1A enzymes, ALDH1A3 is a critical enzyme in the synthesis of RA and the production of RA, influencing genome-wide expression changes. Unsurprisingly, the effects of ALDH1A3 in cancer have been linked to RA in multiple studies (Figure 1). In breast cancer, ALDH1A3-mediated effects on gene expression changes and tumor growth could be mimicked with RA treatment, which suggested that at least a part of ALDH1A3′s cancer-promoting effects could be due to its production of RA and the RA-mediated gene expression changes [45]. A comparison of the gene expression changes induced by ALDH1A3 versus RA treatment in TNBC cells showed partial overlap is consistent with this hypothesis; however, the partial overlap also suggests there are genome-wide gene expression effects induced by ALDH1A3 in cancer that are independent of RA [89]. Effects of ALDH1A3 connected to RA signaling were also demonstrated in melanoma, where gene expression signatures of ALDH+ melanoma cells include RA-driven target genes harboring RAREs [37]. The survival of mesenchymal glioma stem cells was dependent upon the expression of RA-inducible tissue transglutaminase (TG2) [78].

ALDH1A3 also induces gene expression changes indirectly or independently of RA, leading to effects in cancer. Emerging evidence in recent years has detailed the regulation of miRNAs and lncRNAs by ALDH1A3, with subsequent effects on gene expression. In colon cancer, ALDH1A3 upregulated transcription factor zinc finger E-box binding homeobox 1 (ZEB1) and snail family transcriptional repressor 2 (SNAI2) by inhibiting miR-200 family members, leading to increased invasion [50].

ALDH1A3 and RA induce lncRNA non-coding RNA in the aldehyde dehydrogenase 1A pathway (NRAD1), formerly known as LINC00284 in breast cancer [77]. NRAD1 is primarily nuclear and was shown to bind to chromatin, which was linked to NRAD1 and ALDH1A3-mediated gene expression changes. NRAD1 may be a cause of at least some of the stemness and tumorigenicity associated with ALDH1A3 in breast cancer, as its knockdown reduced tumor growth and mammosphere formation potential. In colorectal cancer, ALDH1A3 was similarly found to induce LINC00284 (NRAD1); however, its cancer-promoting effects were mediated through miRNA interactions [77]. NRAD1/LINC00284 has since been implicated as a crucial regulator of gene expression and tumor progression through sponging various miRNAs in multiple cancers [77,90,91,92,93,94]. 

## 10. Effects of ALDH1A3 on Glycometabolism and Other Metabolic Pathways in Cancer

Cancer cells need altered metabolism to fulfill the extensive energy requirements necessary for malignant growth. The “Warburg effect” describes the effect where cancer cells use glycolysis even in the presence of oxygen (aerobic glycolysis) to generate ATP for cellular functions [95,96,97,98]. In tumors, only a fraction of available glucose is completely metabolized by oxidative phosphorylation and most glucose is converted into lactate. CSCs also display altered energetics [99], and evidence suggests that ALDH1A3 plays a part in the metabolic reprogramming of cancer. 

A few studies indicate a link between ALDH1A3 and the “Warburg effect”, where glycometabolism predominates in the tumor or cancer cells and is associated with ALDH1A3 and cancer progression. ALDH1A3 promotes pancreatic cancer progression and metastasis by increasing cellular glycolysis [52]. Mechanistically, this effect is mediated by ALDH1A3 increasing expression of glycolysis enzymes, including hexokinase 2 (HK2) [52]. ALDH1A3-mediated glycolysis and induced expression of HK2 is indirect and governed by its activation of the PI3K/AKT/mTOR pathway and subsequent increased expression of nuclear hormone peroxisome proliferator-activated receptor gamma (PPARγ) [52]. PPARγ induces the expression of HK2, which has a PPAR response element in its promoter. Mesenchymal glioblastoma stem cells also demonstrate increased glycolytic activity associated with ALDH1A3 expression [38]. The growth reduction of the glioblastoma stem cells followed by ALDH1A3 inhibition demonstrates the tumor-promoting effects of ALDH1A3 connected to glycolysis [38]. Similarly, another study reported that inhibiting ALDH1A3 in glioblastoma cells also reduced glycolytic activity, invasion, and tumor growth [95]. 

In addition to glycometabolism, there is evidence that other metabolic pathways are also dysregulated by ALDH1A3 in cancer. Expression changes induced by ALDH1A3 in TNBC cells (e.g., upregulated 4-aminobutyrate aminotransferase (ABAT) and downregulated glutamate decarboxylase 1 (GAD1)) results in dysregulated metabolism of γ-aminobutyric acid (GABA) [96]. This dysregulation of GABA metabolism was connected to ALDH1A3-mediated metastasis of MDA-MB-231 cells to the lungs and brains of mice.

## 11. Role of ALDH1A3 in Type 2 Diabetes

Dysregulated ALDH1A3 also has effects on other pathologies, including type 2 diabetes. Glucose homeostasis is maintained by the balance of insulin and glucagon in the blood [43]. Insulin is produced by the β-cells of the pancreas and insulin production is increased to meet the metabolic demands. Chronic metabolic demand is associated with obesity invariably which results in declined β-cell function and reduced mass from cell death and dedifferentiation. Upon dedifferentiation, β-cells assume a progenitor-like state and fail to produce insulin. Together, insulin resistance and β-cell impairment result in dysregulated glucose control and the development of type 2 diabetes. 

Dedifferentiated β cells have increased ALDH1A3 expression compared to normal β-cell [100]. High ALDH1A3 in β-cells was also correlated with decreased insulin in islets and dedifferentiating prompting precursors, including MAF BZIP transcription factor A (MafA) and NK6 homeobox 1 (Nkx6) [97]. ALDH1A3 overexpression in β-cells is associated with loss of transcription factor, Forkhead box protein O1 (FOXO1), a key regulator of glucose homeostasis. When FOXO1 levels decrease in β-cell, ALDH1A3 levels increase by 30-fold, allowing for it to cause mitochondrial dysfunction without alerting oxidative stress [97]. ALDH1A3 expression is also suppressed by miR-483 in β cells [98]. 

There is evidence that inhibiting ALDH1A3 expression in β-cells could restore insulin levels. A diet combined with red yeast rice, bitter gourd, and chromium led to reduced ALDH1A3 and an increase in FOXO1 levels in diabetic mice [101]. Another study utilized O304, which is a pan-AMP-activated protein kinase (AMPK) activator, to restore chromatin marks and inhibit ALDH1A3 expression, resulting in increased insulin levels [102].

## 12. The Role of ALDH1A3 in Cardiac Function and Pathology

ALDH1A3 is the main contributor to ALDH activity in cardiac progenitor cells [103], and cardiac progenitor cells from atrial appendages can be isolated based on ALDH activity [104]. These cells retained the differentiation potential when injected into infarcted hearts [105]. This finding shows promise for the role of ALDH1A3 in cardiovascular disease therapy against reperfusion injury.

A recent study reports ALDH1A3 is the most highly upregulated metabolic gene in pulmonary arterial hypertension [106]. Mice lacking ALDH1A3 are resistant to developing pulmonary arterial hypertension and ALDH1A3 regulates mRNA expression of cell cycle and metabolic genes involved in pulmonary arterial hypertension that is necessary for ALDH1A3-dependent proliferation and glycolysis [106]. 

## 13. Targeting CSCs and ALDH1A3

Targeting CSCs by inhibiting CSC-associated pathways, markers, proteins, and non-coding RNAs are common strategies being pursued. For example, the possibility of targeting EpCAM with anti-EpCAM antibodies has been explored extensively and reviewed elsewhere [107]. The Notch signaling pathway, which is commonly activated in CSCs across cancer types, is also a highly explored strategy for targeting CSCs [108]. In particular, γ-secretase inhibitors, which inhibit Notch receptor proteolytic cleavage and signaling, have demonstrated preclinical efficacy with induction of CSC differentiation and apoptosis, inhibition of EMT, and sensitizing tumors to chemotherapies [109]. Targeting non-coding RNAs enriched in CSCs with antisense oligonucleotides has also been suggested as a possibility [85]. The possibility of inhibiting ALDHs and ALDH1A3 specifically in the treatment of cancer and targeting of CSCs has also been investigated in recent years by various drugs.

Many compounds have general or semi-specificity for inhibition of ALDH isoforms. These compounds include DEAB, chloral hydrate, citral, coprine, diadzin, gossypol, pargyline, and disulfiram [3]. Disulfiram is an old drug—it has been used to treat alcohol abuse for over 70 years [110]. The liver enzyme alcohol dehydrogenase converts alcohol to acetaldehyde, which then becomes converted into non-toxic acetic acid by liver ALDH1A1 and ALDH2 [110]. Disulfiram inhibition of liver ALDH1A1 and ALDH2 leads to toxic accumulation of acetaldehyde, resulting in an adverse reaction to alcohol consumption that psychologically conditions the patient to associate alcohol with physical pain. Work has been conducted to repurpose this classic anti-alcoholism drug as a possible treatment for various cancers [111].

Disulfiram works as an anti-cancer agent by several potential mechanisms; in addition to inhibiting ALDHs, it inhibits proteasome function (when complexed with copper; CuET), E3 ligases, and intriguingly may also be a DNA-demethylating agent [112,113]. In terms of inhibiting the ALDH1A3 isoform specifically, disulfiram has minimal ALDH1A3 targeting activity in breast cancer cells [61] and inhibited glioblastoma stem cells independent of effects on ALDH1A3 [114]. However, the disulfiram copper complex CuET inhibited colorectal cancer progression by downregulating ALDH1A3 gene expression [62]. 

Some specificity for ALDH1A3 was observed in citral, where µM concentrations inhibited Aldefluor-mediated ALDH1A3 activity in breast cancer cells and encapsulated citral inhibited ALDH1A3-mediated breast tumor growth [61]. In contrast, the same study showed that diadzin, chloral hydrate, coprine, gossypol, and pargyline did not inhibit ALDH1A3 activity of breast cancer cells even at 100 µM concentrations. Interestingly, a modified diadzin analog synthesized to inhibit ALDH1A3 (i.e., imidazo [1,2-*a*] pyridine, G11), had in vivo efficacy in a glioblastoma tumor model [81]. In cell-free assays of ALDH1A3 activity, G11 had half maximal inhibitory concentration (IC50) of 22.8μM. A further modification of G11 generated MF-7, which demonstrated improved IC50 in cell-free assays (4.7μM) [115]. MF-7 treatment increased the survival of mice in a breast cancer brain metastasis model [115]. The later derived analog NR-6 showed similar anti-cancer activity [116]. More recently, the type 5 phosphodiesterase (PDE5) inhibitor E4021 was found to bind to ALDH1A3 by protein affinity chromatography approach and sub-µM amounts of derivatized compound ER-001135935 specifically inhibited ALDH1A3 activity in vitro [117]. 

The solving of the crystal structure of ALDH1A3 complexed with NAD+ and ATRA in 2016 [118] allows for rationally designed ALDH1A3 specific inhibitors that prevent pocket binding of the substrate binding. This was recently demonstrated by the generation of in silico-designed MCI-INI-3, which inhibits ALDH1A3 specifically (IC50 = 0.46 µM) [119]. Although not tested yet for anti-cancer activity, this rationally designed inhibitor has the highest specificity and activity among thus far reported ALDH1A3 inhibitors. The crystal structure of ALDH1A3 also allows for the screening of potential ALDH1A3 inhibitors among library compounds by molecular docking modeling. These in silico analyses identified YD1701 (dibenzo-30-crown10-ether), as an ALDH1A3 inhibitor [120]. It has a reported IC50 of 12.0824 µg/mL (which is equivalent to 22.5 µM based on a molecular weight of 536.63 g/mol). YD1701 inhibited the invasion of colon cancer cells and prolonged the survival of mice implanted with colon cancer xenografts.

Likely, if the clinical use of targeting ALDH1A3 is to be realized, it will be in combination with chemotherapies, immunotherapies, and other adjuvant therapies. Potential combination strategies also include inhibiting ALDH1A3 alongside other drugs that target different CSC markers and pathways to limit the emergence of therapy-resistant CSCs.

## 14. Conclusions

In this review, we have presented the most comprehensive and updated overview of ALDH1A3. ALDH1A3′s role as a CSC marker is well established. Generalities that arose from our ALDH1A3 literature review suggest it has pro-tumor growth and metastatic effects in cancer. Mechanism studies suggest that ALDH1A3-mediated cancer progression and chemoresistance are, in general, due to ALDH1A3-mediated gene expression effects. Gene expression changes induced by ALDH1A3 were often connected to RA signaling; however, there are more recent studies describing gene expression regulation via miRNAs or lncRNAs. Given its varied functional roles, it is of no surprise that ALDH1A3 is implicated in multiple cancers and has prognostic relevance. Beyond cancer, we have highlighted the role of ALDH1A3 in other diseases. Being a metabolic enzyme, ALDH1A3′s association with metabolic diseases, including diabetes, is predictable and there is growing evidence of its effects on glycometabolism.

The evidence suggests that ALDH1A3 could be a novel therapeutic target in various diseases, and we have detailed the inhibitors of ALDH1A3 that have been reported thus far. While no ALDH1A3 inhibitors have been pursued in clinical trials yet, it is a likely future possibility with the increasing specificity of inhibitors being designed and showing preclinical efficacy.

## Figures and Tables

**Table 1:** Incidence and types of ALDH1A3 mutations in cancers that have been reported to be affected by ALDH1A3 expression. The information and the patient datasets were accessed and obtained via cBioPortal.

Cancer Dataset	Patient Number	Amplification	Missense Mutations	Deep Deletion	Truncation Mutation
Prostate (TCGA, Firehose Legacy)	489	0.2%	0.2%	0.2%	0.0%
Prostate Metastasis (SU2C/PCF Dream Team, PNAS 2019)	429	1.4%	0.2%	0.9%	0.0%
Colorectal (TCGA, Firehose Legacy)	220	0.45%	1.8%	0.0%	0.45%
Breast (TCGA, Cell 2015)	816	3.2%	0.5%	0.4%	0.1%
Triple Negative Breast Cancer (TCGA, Cell 2015)	82	9%	0.0%	0.0%	0.0%
Breast Metastasis (Provisional, December 2021)	301	9.3%	0.0%	2.3%	0.0%
Pancreatic (TCGA, Firehose Legacy)	149	2.7%	1.3%	0.0%	0.0%
Glioblastoma (CPTAC, Cell 2021)	96	2.1%	0.0%	0.0%	0.0%
Melanoma (TCGA, Cell 2015)	344	0.0%	0.6%	0.0%	0.0%
Melanoma Metastasis (DFCI, Science 2015)	110	6.3%	1.8%	0.9%	0.0%
Lung (TCGA, Firehose Legacy)	230	1.7%	0.0%	0.4%	0.0%
Lung Never Smokers (NCI, Nature Genetics 2021)	232	0.0%	0.4%	0.0%	0.0%
Non-Small Cell Lung (TRACERx, NEJM & Nature 2017)	100	0.0%	1.0%	0.0%	1.0%
Bile Duct (TCGA, Firehose Legacy)	35	0.0%	0.0%	0.0%	0.0%
